# Effects of different depth of anesthesia on perioperative inflammatory reaction and hospital outcomes in elderly patients undergoing laparoscopic radical gastrectomy

**DOI:** 10.1186/s12871-022-01854-8

**Published:** 2022-10-25

**Authors:** An-qing Lv, Li-cai Huang, Wei-long Lao, Qi-liang Song, Qi-fu Zhou, Zong-ming Jiang, Zhong-hua Chen

**Affiliations:** 1 Department of Anesthesia, Shaoxing People’s Hospital, No. 568 Zhongxing North Road, Yuecheng District, 312000 Shaoxing, China; 2 Department of Anesthesia, Shaoxing University School of Medicine, 312000 Shaoxing, China

**Keywords:** Depth of anesthesia, Closed-loop target control, Inflammatory factors, Bispectral index, Elderly patients

## Abstract

**Background:**

To investigate the effect of different depth of anesthesia on inflammatory factors and hospital outcomes in elderly patients undergoing laparoscopic radical gastrectomy for gastric cancer, in order to select an appropriate depth of anesthesia to improve the prognosis of patients undergoing surgery and improve the quality of life of patients.

**Methods:**

A total of 80 elderly patients aged 65 and above who underwent laparoscopic radical gastrectomy in our hospital were by convenience sampling and randomly divided into two groups : 55 groups ( group H ) and 45 groups ( group L ), 40 cases in each group. The depth of anesthesia was maintained using a closed-loop target-controlled infusion system: the EEG bispectral index was set to 55 in the H group and 45 in the L group. Venous blood samples were collected 2 h (T2), 24 h (T3) and 72 h (T4) after the start of surgery. The intraoperative dosage of propofol and remifentanil, operation duration, postoperative PACU stay time, intraoperative consciousness occurrence, postoperative hospital stay and postoperative pulmonary inflammatory events were recorded.

**Results:**

The patient characteristic of the two groups had no statistical difference and were comparable (*P* > 0.05). The intraoperative dosage of propofol in group H was lower than that in group L (*P* < 0.05). Compared with the L group, the plasma IL-6 and IL-10 concentrations in the H group were significantly increased at T2 (*P* < 0.05), and the plasma IL-10 concentration was significantly increased at T4 (*P* < 0.05). The plasma concentrations of IL-6 and IL-10 were higher in both groups at T2, T3 and T4 than at T1, while at T4, the concentration of TNF-α in group H was higher than at T1 (*P* < 0.05).

**Conclusion:**

When the BIS value of the depth of anesthesia is 45, the perioperative release of inflammatory factors in elderly patients with laparoscopic radical gastrectomy for gastric cancer is less than BIS 55, and does not affect the prognosis.

## Background

The trauma caused by the perioperative period can cause a strong stress response in the patient. The massive release of inflammatory factors is one of the main manifestations of stress response, which can cause adverse effects on the heart, lungs, brain and other organs, especially for elderly patients, excessive inflammatory factors will increase postoperative complications and mortality[1]. This study selected three representative inflammatory factors: IL-6, IL-10 and TNF-α to explore. IL-6 is the most potent endogenous inflammatory mediator that triggers inflammatory responses. IL-10 is an anti-inflammatory cytokine that limits the inflammatory response. TNF-α is one of the earliest inflammatory factors released by the body during traumatic stress, and it is also one of the main factors causing organ damage. Good intraoperative anesthesia depth and sedation management can effectively reduce the perioperative stress response and improve the prognosis of patients[2]. The Bispectral Index (BIS) is an analytical metric based on electroencephalographic (EEG) parameters that is specifically designed to measure patient response during sedative administration [3]. It is a real-time continuous and anesthesia depth detection method considering the nonlinear characteristics of EEG signals, which is convenient and feasible, and optimizes the way of administration. Studies have shown that EEG BIS values between 40 and 60 are acceptable for surgical operations, and can avoid the occurrence of delayed intraoperative consciousness and anesthesia awakening [4]. However, this range is wider. Especially in the anesthesia of elderly patients, a more appropriate depth of sedation is required. At present, there are few researches at home and abroad. Therefore, this study intends to use the CLTCI method under the monitoring of BIS to investigate the effects of different intraoperative sedation depths on perioperative inflammatory factors and prognosis in elderly patients with laparoscopic radical gastrectomy for gastric cancer. The results of this study can provide reference for clinical management.

## Methods

### Study subjects and groups

A total of 80 elderly patients aged 65 and above who underwent laparoscopic radical gastrectomy in our hospital were by convenience sampling and randomly divided into two groups : 55 groups ( group H ) and 45 groups ( group L ), 40 cases in each group. All patients had no history of mental illness, no history of endocrine disease or immune system dysfunction, no liver and kidney dysfunction, no long-term use of hormones or immunomodulatory drugs, sedatives or antidepressants, and no history of alcoholism or drug dependence. There was no significant difference in baseline data such as gender and age between the two groups (*P* > 0.05), which was comparable. Four patients in group H and three patients in group L were excluded due to changes in intraoperative surgical approach or serious postoperative complications. This study was approved by the Ethics Committee of Shaoxing People’s Hospital, and informed consent was obtained from patients and their families (clinical trial registration number: NCT05173909(30/12/2021)).

### Research methods

The patients were visited before the operation, fasting for more than 6 h, no drinking for more than 2 h, and no preoperative medication. After the patient enters the operating room, routine ECG monitoring, upper extremity intravenous infusion, left radial artery puncture and catheterization under local anesthesia to monitor invasive blood pressure, and BIS monitor (Aspect Medical Systems, USA) are connected to monitor the BIS value. Closed-loop target-controlled infusion (CLTCI) with feedback signal control performance, the rate of administration is automatically controlled according to the feedback to achieve the intravenous injection method that maintains the plasma target concentration or the target concentration of the effect chamber[5]. Both groups received closed-loop propofol targeted control, the BIS monitor signal input terminal was connected to the patient, and the output terminal was connected to the EEG monitoring TCI syringe pump. (Beijing Silugao Medical Technology Co., Ltd., China). The propofol on the syringe pump is connected to the patient’s venous access to form a closed-loop target control. Anesthesia induction: midazolam 0.02 mg•kg-1, atracurium cisbesilate 0.3 mg•kg-1, etomidate 0.2～0.3 mg•kg-1, sufentanil 0.5 µg•kg- 1. Perform mechanical ventilation after tracheal intubation, adjust tidal volume to 8–10 mL•kg-1, respiration rate 12–14 times/min, inhalation-expiration ratio 1:2, maintain EtCO2 at 35-40mmHg, adjust oxygen flow rate 2 L • min-1. Anesthesia maintenance: Both groups of patients received intravenous infusion of remifentanil 0.1～0.3 µg•kg-1 min-1 and closed-loop target-controlled infusion of propofol. The BIS of patients in group H was set at 55, and the BIS of patients in group L was set at 45, and atracurium cisbesilate was added intermittently to maintain muscle relaxation. Intraoperative invasive arterial blood pressure is controlled within 25% of the baseline (before the day of surgery), and the heart rate is controlled at 50–90 beats/min. If blood pressure exceeds this target range, phenylephrine and nitroglycerin are regulated. Use isoproterenol and esmolol to regulate heart rate. At the end of the operation, a 48-hour self-controlled intravenous analgesia pump (PCIA) was performed, with ondansetron 16 mg, sufentanil 2 µg/kg, and flurbiprofen axetil 200 mg diluted with normal saline to 100 ml. After the operation, the patients was admitted to the post anesthesia care unit (PACU) for anesthesia resuscitation.

### Observation index

Venous blood samples were collected immediately before the operation (T1), 2 h after the operation (T2), 24 h (T3) and 72 h (T4) after the operation. Plasma interleukin(IL-6), (IL)-10 and tumor necrosis factor(TNF) concentration were determined by enzyme-linked immunosorbent assay. The intraoperative dosage of propofol and remifentanil was recorded, and the postoperative PACU stay time, intraoperative awareness of occurrence, postoperative hospital stay, and postoperative lung inflammation were recorded.

### Statistical methods

SPSS 22 statistical software was used for analysis. The count data is expressed as a percentage, and the chi-square test or Fisher’s exact test is used to test. The measurement data that obeyed the normal distribution were expressed as mean ± standard deviation $(\bar x \pm s)$, and repeated measures analysis of variance was used for different time points and within-group comparisons. The measurement data of non-normal distribution is represented by M (Q1, Q3), and the Wilcoxon rank sum test is used for comparison between groups. *P* < 0.05 indicates that the difference is statistically significant.

## Result

According to the requirements, 80 subjects were included and divided into 2 groups (H group L), 40 cases for each group. The BIS was set to 55 for patients in group H and to 45 for patients in group L. According to the preset method, some patients were excluded due to changes in the surgical approach or serious complications after the trial, and only 73 subjects were actually analyzed, 36 in group H and 37 in group L. The following are the results obtained from the experimental data.

### Patient baseline characteristics

There was no statistical difference in the patient characteristic of the two groups, and they were comparable (*P* > 0.05). (See Table 1)

**Table 1:** Comparison of perioperative general conditions between two groups($\overline x$ ± s, M(QR))

Variable	Group H(*n* = 36)	Group L(*n* = 37)	*P*-Value
Gender:Male/Female	9/27	11/26	0.651
Age	75.17 ± 6.70	73.08 ± 5.37	0.146
Height (cm)	162.36 ± 7.42	164.51 ± 6.56	0.193
Weight (kg)	58.14 ± 9.40	60.46 ± 9.36	0.294
PACU stay time(min)	59.00 (25.00)	58.00 (35.50)	0.712
Operation time(min)	206.50 (57.50)	215.00 (95.00)	0.787
Postoperative hospitalization time(d)	11.00 (3.00)	10.00 (2.50)	0.123
Postoperative pulmonary complications(n(%))	9 (22.50)	5 (13.51	0.343
Intraoperative awareness(n(%))	0	0	1

### Patient outcome

There was no significant difference in operation time, anesthesia recovery time, postoperative hospital stay and postoperative pulmonary complications between the two groups (*P* > 0.05). (see Table 2)

**Table 2:** Comparison of operation time, PACU stay time, intraoperative awareness of occurrence, postoperative hospital stay and postoperative pulmonary complications between the two groups

Variable	Group H(*n* = 36)	Group L(*n* = 37)	*P*-Value
**Operation time[min, M(Q1,Q3)]**	206.5 (172.3,229.8)	215 (154.5,249.5)	0.79
**PACU stay time[min, M(Q1,Q3)]**	59 (51.0,76.0)	58 (45.0,80.5)	0.71
**Intraoperative awareness [case(%)]**	0(0%)	0(0%)	1.00
**Postoperative hospital stay[Day, M(Q1,Q3)]**	11 (10.0,13.00)	10 (9.0,11.5)	0.12
**Postoperative pulmonary complications[case(%)]**	9 (22.5%)	5 (13.51%)	0.22

### Dosage of remifentanil and prednisone

There was no significant difference in the intraoperative dosage of remifentanil between the two groups (*P* > 0.05); the intraoperative dosage of propofol in group H was lower than that in group L (*P* < 0.05). (see Table 3)

**Table 3:** Comparison of intraoperative anesthetic dosage ($\overline x$ ± s)

Variable	Group H (*n* = 36)	Group L (*n* = 37)	*P*-Value
**Propofol**	793.22 ± 294.84^a^	1001.51 ± 431.96	0.019
**Remifentanil**	0.94 ± 0.33	0.94 ± 0.40	0.978

a: compared with L group, *P* < 0.05. Both Propofol and Remifentanil doses are mg

### Inflammatory factors in patients

The plasma levels of IL-6 (396.15 vs. 165.97, *P* < 0.001) and IL-10 (18.05 vs. 10.18, *P* < 0.001) at T2 and IL-10 (5.26 vs. 3.98, *P* < 0.001) at T4 were significantly higher in group H than in group L, and the differences were statistically significant. There was no significant difference in plasma TNF-α concentration between the two groups at each time point (*P* > 0.05).

**Fig. 1:**
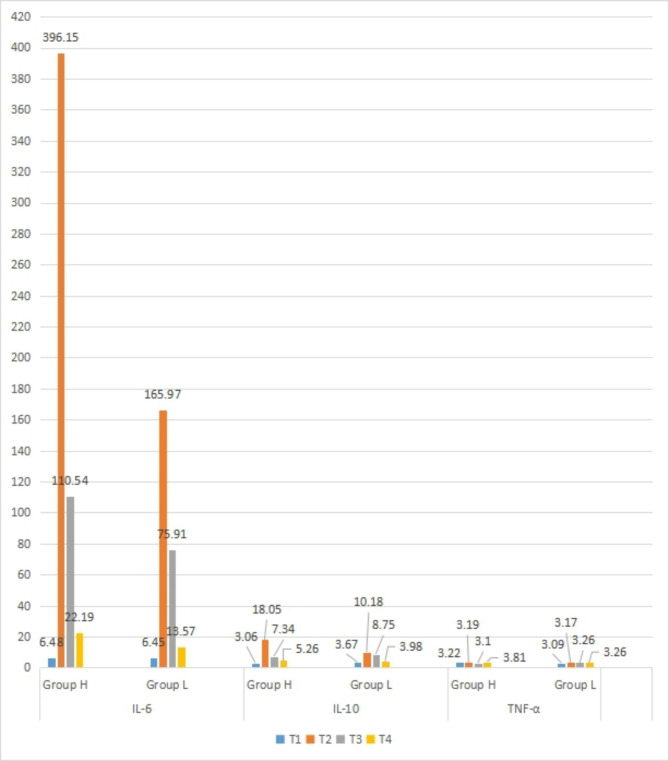
Comparison of inflammatory factors [pg/ml] between group H (n = 36) and group L (n = 37) patients

In-group comparison, the plasma IL-6( 396.15 vs. 6.48; 110.54 vs. 6.48; 22.19 vs. 6.48) and IL-10 (18.05 vs. 3.06; 7.34 vs. 3.06; 5.26 vs. 3.06) concentrations of the two groups at T2, T3 and T4 were higher than those at T1, and the difference was statistically significant (*P* < 0.05). At T4, TNF-α concentration was higher than its T1 level(3.81 vs. 3.22), and the difference was statistically significant (*P* < 0.05). There was no significant difference in TNF-α concentration at each time point in group L (*P* > 0.05). ( Fig. 1)

## Discussion

The effects of different depths of anesthesia on brain tissue metabolism, blood flow, and inflammatory factors are quite different. The depth of anesthesia is generally considered to be a risk factor for postoperative cognitive dysfunction(POCD). As the depth of anesthesia increases, the brain metabolism rate and blood flow decrease and increase the risk of POCD after surgery. Inflammatory factors and related signaling pathways are related to postoperative cognitive impairment. Signal pathways such as P123KS are involved in the process of postoperative cognitive dysfunction[6, 7]. As an important means to reflect the depth of intraoperative anesthesia and sedation, BIS monitoring has been approved by the FDA in 1997 as an indicator for monitoring the depth of anesthesia and sedation during the perioperative period. Mathur S et al. [8] defined a BIS value of 50 to 60 as light anesthesia, and 30 to 40 as deep anesthesia. In this study, the BIS value of deep anesthesia was positioned as 45, and the definition of light anesthesia was 55. However, because the BIS value changes dynamically, it is difficult to maintain BIS at a certain value in conventional anesthesia management methods. The closed-loop target-controlled infusion system used in this study is an anesthesia sedation control system that uses the real-time value of the BIS monitor to feedback and regulate the drug infusion rate, which can ensure that the depth of anesthesia [9] is accurate to a specific value.There has been a recent study [10] of the effects of different depth of anesthesia under closed-loop target-controlled infusion on cognitive function and adipocytokine and oxidative stress responses in elderly surgical patients. In this study, the BIS value of the target control pump in the deep anesthesia group was set to 40, and the BIS value of the target control pump in the light anesthesia group was set to 50. Finally, it is concluded that the selection of moderate deep anesthesia for elderly surgical patients under closed-loop target-controlled infusion can reduce the incidence of POCD to a certain extent and facilitate the postoperative rehabilitation of patients. This shows that the closed-loop system can be used for the input of surgical anesthesia.

Surgery and other invasive procedures routinely performed during general anesthesia may cause inflammation in patients. In these cases, immune system activation is not always beneficial to the patient, and may cause host cells, tissues, and even the entire organ system concomitanted risk of harmful effects[11]. In this study, three representative inflammatory factors-IL-6, IL-10, and TNF-α were selected. IL-6 is the strongest endogenous inflammatory mediator that initiates the inflammatory response. It generally starts to increase 30 min after being stimulated, and the peak appears at 4 to 6 h, and the increase is consistent with the degree of stress caused by surgery[12]. IL-10 is an anti[13]-inflammatory cytokine, which limits the inflammatory response and plays a protective role in the inflammatory response caused by traumatic stress. TNF-α is one of the earliest inflammatory factors released by the body when subjected to traumatic stress, and it is also one of the main factors that cause organ damage. It can activate a variety of inflammatory mediators, cause a cascade of amplification reactions, and aggravate organ damage[14]. The results of this study showed that plasma IL-6 and IL-10 concentrations increased significantly 2 h after the start of the operation, and fell back 24 h after the operation. And 2 h after the start of the operation, the plasma IL-6 and IL-10 concentrations in group H were significantly higher than those in group L. At the same time, the plasma IL-6 and IL-10 concentrations of patients in group H were significantly higher than those in group L 2 h after the start of the operation. This is similar to the results of Hou et al.[15], suggesting that the depth of anesthesia and sedation with an intraoperative BIS value of 45 can reduce the release of inflammatory factors. The possible reasons are as follows: First, Quan and other [4] studies have shown that the average arterial pressure, heart rate, and blood catecholamine concentration of patients during light anesthesia are higher than those during deep anesthesia. Therefore, compared with deep anesthesia, the body’s stress response is greater during light anesthesia under the same stimulation, which leads to a stronger inflammatory response; second, the results of this study show that the amount of propofol during operation in group L was significantly greater than Group H. Studies have shown that propofol can inhibit the inflammatory response [16] through the NF-κB pathway. At 72 h after operation (T4), the plasma TNF-α concentration in group H was significantly increased, and at this time, the plasma IL-10 concentration in group H was also significantly higher than that in group L. This may be related to postoperative pain. Because the PCIA pump configured in this study is a 48-hour dose, after the analgesic pump is stopped, the patient’s pain will increase compared to before, and the painful stress will increase the release of inflammatory factors. And because the inflammatory reaction in the L group was better controlled than that in the H group, the above results appeared. In this study, there was no significant difference in the postoperative PACU resuscitation time and postoperative hospital stay between the two groups. Although there was no statistically significant difference in the incidence of postoperative pulmonary complications between the two groups, the incidence of patients in group H was slightly higher than that in group L. Combined with the results of previous studies, it may be necessary to further expand the sample size.

The shortcoming of this study is that multiple target BIS values are not set, so it is impossible to determine the most appropriate depth of anesthesia and sedation for elderly patients undergoing laparoscopic radical gastric cancer treatment. The length of anesthesia, the amount of fluid, etc., are also not considered in the influencing factors of stress response, which will have an impact on the accuracy of our findings. These will be further improved in future studies.

## Conclusion

In summary, when the BIS value of the depth of anesthesia and sedation is 45, the perioperative release of inflammatory factors in elderly patients undergoing laparoscopic radical gastrectomy is less, and the prognosis is not affected at the same time.

## Data Availability

All data generated or analyzed during this study are included in this published article.
